# A retrospective study of central nervous system shunt infections diagnosed in a university hospital during a 4-year period

**DOI:** 10.1186/1471-2334-6-43

**Published:** 2006-03-08

**Authors:** Suzan Sacar, Huseyin Turgut, Semra Toprak, Bayram Cirak, Erdal Coskun, Ozlem Yilmaz, Koray Tekin

**Affiliations:** 1 Department of Infectious Diseases and Clinical Microbiology, Pamukkale University, Faculty of Medicine, Denizli, Turkey; 2 Department of Neurosurgery, Pamukkale University, Faculty of Medicine, Denizli, Turkey; 3 Department of General Surgery, Pamukkale University, Faculty of Medicine, Denizli, Turkey

## Abstract

**Background:**

Ventriculoperitoneal (VP) shunts are used for intracranial pressure management and temporary cerebrospinal fluid (CSF) drainage. Infection of the central nervous system (CNS) is a major cause of morbidity and mortality in patients with CSF shunts. The aim of the present study was to evaluate the clinical features, pathogens, and outcomes of 22 patients with CSF shunt infections collected over 4 years.

**Methods:**

The patients with shunt insertions were evaluated using; age, sex, etiology of hydrocephalus, shunt infection numbers, biochemical and microbiological parameters, prognosis, clinical infection features and clinical outcome.

**Results:**

The most common causes of the etiology of hydrocephalus in shunt infected patients were congenital hydrocephalus-myelomeningocele (32%) and meningitis (23%). The commonest causative microorganism identified was *Staphylococcus (S.) aureus*, followed by *Acinetobacter spp*., and *S. epidermidis*.

**Conclusion:**

In a case of a shunt infection the timely usage of appropriate antibiotics, according to the antimicrobial susceptibility testing, and the removal of the shunt apparatus is essential for successful treatment.

## Background

Ventriculoperitoneal shunts are used for maintaining a specific intracranial pressure and generally permanent, but on occasion temporary CSF drainage [1-3]. The placement and revision of VP shunts remains a mainstay in the surgical treatment of hydrocephalus [4]. Infection is the foremost complication of CSF shunt implantation [1,2,4,5]. The incidence of CSF infection secondary to ventriculostomy, shunt insertions has been quoted in previous reports as being between 2.2% and 39 % [1,3]. Many factors have been reported to be associated with increased risk of infection, including the age of patient, etiology of hydrocephalus, the type of shunt implanted, and the surgeon's experience [2]. The treatment of shunt infections requires not usually extension of the hospital stay by 2 or 3 weeks and additional surgery [2,6].

The aim of the present study was to evaluate the clinical features, pathogens, and outcomes of 22 patients with cerebrospinal fluid (CSF) shunt infections collected over 4 years.

## Methods

The study protocol was approved by the Pamukkale University Research Ethics Committee, Denizli, Turkey, and it complied with the principles outlined in the Helsinki Declaration [7]. Written informed consent was obtained from each patient or a legally designated representative.

The clinical notes of 124 consecutive patients having CNS shunt placement operation for various etiology between 2000 December and 2004 December were reviewed of Pamukkale University, Medical School, Department of Neurosurgery. Operations were performed by two neurosurgeons. One of them (B.C.) has a subspecialty on pediatric neurosurgery. The patients with shunt insertions were analyzed according to; age, sex, etiology of hydrocephalus, shunt infection numbers, prognosis, clinical infection features and clinical outcome. Biochemical and microbiological parameters were evaluated from the patients' clinical notes and the notes of a specialist in Infectious Diseases.

To define the shunt infected group, at least one of the following criteria was present: 1) the presence of an organism isolated from CSF culture; 2) the presence of fever (>38,5°C) in the absence of other recognized causes, with institution of appropriate antimicrobial treatment and any of the following; increased white cell count (> 50% polymorphonuclear leucocytes), increased protein and/or decreased glucose (< 15 g/dl) in CSF, or organisms visible on CSF Gram stain. While CSF infections within the initial 30 days of ventricular catheter insertion were considered to be early, the infections which occur 30 days after the insertion were considered as late.

Only one dose prophylactic antibiotic (cefuroxime or ampicillin) was given perioperatively to the patients.

## Results

Shunt placement operations in 124 patients had been made in the investigation period. Twenty-six (20.9 %) of these 124 patients were younger than two years old. The median age of adult patients were 46 (7–72). The type of the shunt used was a medium pressure type shunt (burr-hole valve medium pressure, from Medtronic, California, USA). Etiological profile of CNS shunt placement operations of 124 patients is exhibited in Table 1 Among these 124 patients, 22 (17.7 %) patients were determined as shunt infected. Thirty shunt infection attacks were confirmed in these 22 patients. The group consists of 12 males and 10 females. Ten of the 22 patients were younger than two years old. The remaining adult patients' average age was 44.3 years. Clinical data of the 22 patients were designated in Table 2. In our study group, while CSF infections of 14 patients were seen in the early period, infections of remaining 8 patients were seen in the late period. Some of the children had another site infection (urinary tract infection, bronchitis, etc.) and majority of adult patients with comorbidities had hypertension, diabetes mellitus and another site infection (urinary tract infection, pneumonia). During the study, *Acinetobacter *has been isolated in both CSF and tracheal aspirates simultaneously in two patients. Similarly, *E. coli *in two patients and *Klebsiella pneumoniae *in one patient have been isolated in both urine and CSF. The most common causes of the etiology of hydrocephalus in shunt infected patients were congenital hydrocephalus- closed myelomeningocele (32%) and meningitis (23%); than respectively intracranial mass (23%), intracranial hemorrhage (9%), trauma (9%) and intracranial abscess (4.5%). The intracranial abscess was localized on supratentorial region and the causative microorganism was not identified.

**Table 1:** Etiological profile of CNS shunt placement operations of 124 patients

Etiology of hydrocephalus	No. of Patients (%)
Congenital hydrocephalus-myelomeningocele	18 (14.6)
Meningitis- hydrocephalus	22 (17.8)
Intracranial mass	28 (22.5)
Intracranial hemorrhage	16 (12.9)
Trauma	11 (8.8)
Intracranial abscess	8 (6.5)
Other	21 (16.9)
Total	124 (100)

**Table 2:** Clinical data of the patients

	Age-sex	Etiology of hydrocephalus	Interval between surgery and development of infection	Attack number	Outcome
1	12 month – F	Post meningitic hydrocephalus	5 day	2	Exitus
2	14 month – F	Intracranial mass	4 month	1	Good
3	4 month – M	Congenital hydrocephalus-myelomeningocele	16 day	1	Good
4	2 month – M	Congenital hydrocephalus-myelomeningocele	3 day	2	Exitus
5	7 month – M	Congenital hydrocephalus-myelomeningocele	3 month	2	Good
6	3 month – F	Congenital hydrocephalus-myelomeningocele	4 day	1	Good
7	1 month – F	Congenital hydrocephalus-myelomeningocele	38 day	1	Good
8	10 month – F	Congenital hydrocephalus-myelomeningocele	4 month	1	Good
9	18 month -M	Congenital hydrocephalus-myelomeningocele	45 day	2	Good
10	5 month – M	Post meningitic hydrocephalus	8 day	2	Good
11	24 y – M	Trauma	7 day	2	Good
12	36 y – F	Trauma	3 day	1	Good
13	65 y – M	Intracranial hemorrhage	12 day	1	Good
14	56 y – F	Intracranial hemorrhage	2 month	1	Good
15	15 y – M	Post meningitic hydrocephalus	21 day	1	Good
16	33 y – F	Post meningitic hydrocephalus	5 day	1	Good
17	7 y – M	Post meningitic hydrocephalus	11 day	1	Good
18	72 y – M	Intracranial abscess	13 day	2	Good
19	45 y – M	Intracranial mass	17 day	1	Good
20	59 y – M	Intracranial mass	15 day	2	Exitus
21	51 y – F	Intracranial mass	5 month	1	Good
22	69 y – F	Intracranial mass	3 month	1	Good

M: Male, F: Female. y: year

From CSF cultures that were taken during each infection attack in only 20 was a microorganism isolated. The most common causative microorganism identified was *S. aureus*, followed by *Acinetobacter spp*., and *S. epidermidis *(Table 3).

**Table 3:** Microbiologic profile of CSF shunt infections attacks

Organism	Incidence (%)
*Staphylococcus aureus*	6 (30)
*Acinetobacter spp*.	4 (20)
*Staphylococcus epidermidis*	3 (15)
*Pseudomonas aeruginosa*	2 (10)
*Klebsiella pneumoniae*	1 (5)
*Escherichia coli*	1 (5)
*Enterobacter aerogenes*	1 (5)
*Enterococci D group*	1 (5)
*Flavobacterium odoratum*	1 (5)
Total	20 (100)

Up to two-thirds of the shunt infected patients' fevers were high (> 38.3°C). From the CSFs that were taken during the 30 infection events, leucocytes counts were increased in 12.

Each infection was treated with external ventriculostomy drainage (EVD) and intravenous antibiotics. The staphylococcus species were susceptible to oxacillin in 7 cases and the 2 patients with a resistant *S. aureus *strain required glycopeptide therapy. Three patients died from complications of shunt infections and 8 patients had a recurrent shunt infection.

## Discussion

A variety of different shunt systems have been evaluated but ventriculoatrial (VA) and VP shunts have been employed most widely in clinical practice [3]. VP shunting has become the diversion procedure of choice because of its shorter operative time and the need for fewer revisions than VA shunts [3]. Although all shunt implant procedures are associated with a high risk of infection and subsequent mortality [8], the rate of infection does not appear to differ greatly between VA and VP shunts [3].

In our patient' population, the majority of devices were placed for congenital hydrocephalus-myelomeningocele. Many studies suggest that the etiology of hydrocephalus was correlated with infections; however, in our study, the numbers of cases were insufficient to determine this issue [2].

Children are more likely than adults to acquire shunt infection, perhaps because of longer hospital stay, higher skin bacterial concentrations, immature immune systems, or more adherent strains of bacteria [9,3]. The rates of infections that are experienced in infants less than 6 months of age are generally two to three times greater than those observed in older children. However, a higher incidence of shunt infections had also been found in the geriatric population [2]. In this study, the shunt infections appeared to be more frequent in the under 2 year-old population.

Early studies reported rates of CSF shunt infection ranging from 1.5 to 39 %; however, during the past two decades infection rates have dropped to 2–9 % [3]. In a recent study in our country the rate of shunt infections was reported to be 14.5 % [6].

Fever is the most common manifestation of CNS shunt infections. Fever in a patient with CNS shunt should always prompt suspicion of shunt infection. Presentation by other non-specific symptoms such as nausea, vomiting, malaise, headache and meningismus are variable [2,3,6].

Examination of CSF should be performed in all patients with suspected shunt infection. Bacterial and fungal cultures of CSF, in addition to blood culture, should be obtained from these patients. Administration of antibiotics to a patient with suspected shunt infection before obtaining CSF culture reduces the likelihood of obtaining a positive culture [9].

The bacteria responsible for most shunt infections are commensal organisms with low virulence. As it appears that intraoperative contamination by skin flora or airborne skin organisms are the most important mechanism of infection of CSF shunts, efforts have been directed at improving intraoperative asepsis and reducing contamination of the operative field [3]. The organisms most frequently causing infections of indwelling CNS prostheses are the coagulase-negative staphylococci. The second most frequent pathogen is *S. aureus *[2,3,10,11] Previously published microbiologic profile of CSF shunt infections is showed in Table 4. It should not be forgotten that the cause of nosocomial meningitis and shunt infections are predominantly gram-negative bacilli and microorganisms of the Staphylococcus genus [6,10]. Gram negative enteric bacteria and *Pseudomonas spp*. account for about 5–10 % of infections and are associated with greater morbidity and mortality [3]. In our study, the rate of Gram- negative microorganisms was fairly higher It can be speculated that, simultaneous infections in other parts of the body which were caused by the same gram negative microorganisms may be responsible for that higher incidence. Contrary to the literature, where gram positive microorganisms are found to predominate in the shunt infections, in a recent study reported in our country the results are similar to ours; the rate of gram negative and positive microorganisms was approximately equal [6].

**Table 4:** Previously published microbiologic profile of CSF shunt infections^a^

Organism	Incidence (%)
*Staphylococcus epidermidis*	32–70
*Staphylococcus aureus*	12–48
*Streptococcal species*	6–10
Enteric Gram- negative bacilli	6–20
Anaerobes	6

^a ^Pooled data ^3,11,15,16^

Recent studies in our country report that the most frequent isolated organism of nosocomial meningitis is *Acinetobacter baumannii *[12,13]. Our cases had various predisposing factors for resistant hospital acquired microorganisms such as following treatment in the intensive care unit. Colonization with nosocomial pathogens, broad spectrum antibiotic usage and serious underlying disease were also contributory factors. Perioperative prophylaxis is generally targeted on staphylococci. Host defenses were also impaired, however, because of the foreign body nature of the shunt. In the time period of the isolation of multi-drug resistant *Acinetobacter spp*. from CSF culture there was isolation of the same microorganism from multiple sites in many patients in the intensive care unit.

CSF shunt infections are usually difficult to treat with systemic antibiotics alone [3,8]. In our practice the management of infections associated with CNS shunts was usually surgical removal of the shunt, temporary external CSF drainage, parenteral antimicrobial therapy with shunt replacement after the infection had been eradicated. This approach is the same as other authors [3,8,14].

Antimicrobials given intrathecally should be constituted in a preservative- free medium to reduce the risk of arachnoiditis. Thus intraventricular antibiotic therapy should be used only if there is a reason to believe that therapeutic CSF concentrations cannot be achieved as a consequence of severe scarring of the choroid plexus or if the antimicrobial of choice is known to have poor CSF penetration, such as an aminoglycoside [3].

The use of perioperative prophylactics for shunt implantation procedures has been controversial [3,14]. Short-term perioperative antimicrobial prophylaxis may be of benefit in preventing shunt infections [3].

## Conclusion

Infection remains the most serious complication of VP shunt placement. Although the etiology of shunt infections is predominantly gram positive organisms in many centers, our results show that infections with gram-negative bacteria form a significant species percentage in the shunt infection in our center. We recommend that the catheter should be inserted under aseptic techniques and should not be replaced unless it is clinically demonstrated such as CSF shunt dysfunction etc. In case of a catheter infection, it is both necessary to remove the shunt and commence the systemic antibiotic treatment. It should also not be forgotten that the timely usage of appropriate antibiotics according to the antimicrobial susceptibility testing is essential for successful treatment.

## Competing interests

The author(s) declare that they have no competing interests.

## Authors' contributions

SS carried out the microbiological studies, conceived of the study, and participated in its design and coordination. HT and KT participated in the design of the study and wrote the manuscript. ST, BC, EC, OY participated in the collection and clinical evaluation of patients. All authors read and approved the final manuscript.

## Pre-publication history

The pre-publication history for this paper can be accessed here:


